# Surface Ammonia-Oxidizer Abundance During the Late Summer in the West Antarctic Coastal System

**DOI:** 10.3389/fmicb.2022.821902

**Published:** 2022-03-25

**Authors:** María E. Alcamán-Arias, Jerónimo Cifuentes-Anticevic, Beatriz Díez, Giovanni Testa, Macarena Troncoso, Estrella Bello, Laura Farías

**Affiliations:** ^1^Departamento de Oceanografía, Universidad de Concepción, Concepción, Chile; ^2^Center for Climate and Resilience Research (CR)^2^, Santiago, Chile; ^3^Escuela de Medicina, Universidad Espíritu Santo, Guayaquil, Ecuador; ^4^Departamento de Genética Molecular y Microbiología, Pontificia Universidad Católica de Chile, Santiago, Chile; ^5^Center for Genome Regulation (CGR), Universidad de Chile, Santiago, Chile; ^6^Programa de Postgrado en Oceanografía, Departamento de Oceanografía, Universidad de Concepción, Concepción, Chile; ^7^Research Center Dynamics of High Latitude Marine Ecosystems (IDEAL), Punta Arenas, Chile

**Keywords:** nitrification, ammonia-oxidizers, Western Antarctic Peninsula, Archaea, Bacteria, photic layer

## Abstract

Marine ammonia oxidizers that oxidize ammonium to nitrite are abundant in polar waters, especially during the winter in the deeper mixed-layer of West Antarctic Peninsula (WAP) waters. However, the activity and abundance of ammonia-oxidizers during the summer in surface coastal Antarctic waters remain unclear. In this study, the ammonia-oxidation rates, abundance and identity of ammonia-oxidizing bacteria (AOB) and archaea (AOA) were evaluated in the marine surface layer (to 30 m depth) in Chile Bay (Greenwich Island, WAP) over three consecutive late-summer periods (2017, 2018, and 2019). Ammonia-oxidation rates of 68.31 nmol N L^−1^ day^−1^ (2018) and 37.28 nmol N L^−1^ day^−1^ (2019) were detected from illuminated 2 m seawater incubations. However, high ammonia-oxidation rates between 267.75 and 109.38 nmol N L^−1^ day^−1^ were obtained under the dark condition at 30 m in 2018 and 2019, respectively. During the late-summer sampling periods both stratifying and mixing events occurring in the water column over short timescales (February–March). Metagenomic analysis of seven nitrogen cycle modules revealed the presence of ammonia-oxidizers, such as the Archaea *Nitrosopumilus* and the Bacteria *Nitrosomonas* and *Nitrosospira*, with AOA often being more abundant than AOB. However, quantification of specific *amo*A gene transcripts showed number of AOB being two orders of magnitude higher than AOA, with *Nitrosomonas* representing the most transcriptionally active AOB in the surface waters. Additionally, *Candidatus* Nitrosopelagicus and *Nitrosopumilus*, phylogenetically related to surface members of the NP-ε and NP-γ clades respectively, were the predominant AOA. Our findings expand the known distribution of ammonium-oxidizers to the marine surface layer, exposing their potential ecological role in supporting the marine Antarctic system during the productive summer periods.

## Introduction

The dissolved inorganic nitrogen pool that supports marine production is primarily the result of ammonium (NH_4_^+^), nitrite (NO_2_^−^), and nitrate (NO_3_^−^) formation and regeneration *via* different biogeochemical pathways. Nitrification forms regenerated NO_3_^−^ (aerobic oxidation of NH_4_^+^ to NO_2_-and then to NO_3_^−^; Falkowski et al., 2003; Clark et al., 2007), which on a global ocean scale can supply approximately half of the NO3-consumed by phytoplankton, thereby having a significant impact on the nitrogen cycle and biomass production (Fernandez et al., 2005; Martin and Pondaven, 2006).

Particularly in polar regions, the estimated ammonia-oxidation rates vary greatly between summer and winter seasons because ammonia-oxidizers depend on the NH_4_^+^ generated by heterotrophic processes, such as excretion and microbial degradation of organic nitrogen, which in turn are coupled with primary production during the summer (Ward, 2000). In the West Antarctic Peninsula (WAP), high ammonia-oxidation rates (220 nM day^−1^) have been mostly detected during the winter (Tolar et al., 2016), when light-driven primary production and competition from phytoplankton are low (Christman et al., 2011), whereas the summer rates (>24 nM day^−1^) are comparatively low. The diminished ammonia-oxidation rates recorded during the summer (Tolar et al., 2016) could be explained by light-inhibition of ammonia-oxidizers (Guerrero and Jones, 1996), as well as by competition for ammonium with phytoplankton and heterotrophic bacteria during this highly productive period (Ward, 2000).

The first step in nitrification is NH_4_^+^ oxidation to NO_2_-by ammonia-oxidizing bacteria (AOB; Betaproteobacteria and Gammaproteobacteria) and archaea (AOA; Thaumarchaeota). AOB have been cultivated for over 100 years and were essential for the discovery of and early research on chemoautotrophy (Wallace and Nicholas, 1969). Conversely, marine Thaumarchaeota were only discovered two decades ago (DeLong, 1992; Fuhrman and McCallum, 1992; Könneke et al., 2005). Due to limited available isolates, the effect of environmental drivers on the marine activity and distribution of this phylum is still not fully understood. Furthermore, the archaeal *amo*A gene is generally more abundant than the bacterial *amo*A gene in many marine environments (Mincer et al., 2007; Beman et al., 2008; De Corte et al., 2009), where AOA often show a wider distribution that commonly correlates with the ammonia-oxidation rate (Beman et al., 2012).

The Southern Ocean was among the first marine environments where Archaea were detected using molecular techniques (DeLong et al., 1994). In the WAP, the AOA Thaumarchaeota (e.g., *Nitrosopumilus* spp.) reportedly account for up to 16%–34% of the bulk picoplankton community of nearshore surface and deeper seawaters off Anvers Island during the late winter period (DeLong et al., 1994; Murray et al., 1998; Church et al., 2003; Alonso-Sáez et al., 2011; Tolar et al., 2016). These values represent 16% of the prokaryotes in the Antarctic surface waters (0–150 m) and are even higher (up to 26%) in the deeper Circumpolar Deep Waters (CDW; >150 m; Alonso-Sáez et al., 2011; Tolar et al., 2016). However, transition from the winter to the summer season denotes a significant decrease in nitrifying archaeal abundances, particularly at the surface (0–150 m; 1–2% of the prokaryotes) compared to the subsurface (150–250 m, 5–10%) and deep (<250 m; 13–17%) waters (Murray et al., 1998; Church et al., 2003; Alonso-Sáez et al., 2011; Grzymski et al., 2012). In contrast, Bacteria in polar waters show spatiotemporal variability in their abundance and distribution in the WAP (Murray et al., 1998; Church et al., 2003), being relatively prevalent in the summer upper water column (0–100 m, 3.9 × 10^5^ cells ml^−1^) comprising 89% of the total picoplankton assemblage in this depth range, but decreasing to 8.9 × 10^4^ cells ml^−1^ with depth in the CDW (>150 m; Church et al., 2003). Furthermore, AOB such as those represented by *Nitrosospira*-like sequences, have been reported as ubiquitous and important AOB in Arctic and Antarctic samples mostly below 50 m depth (Hollibaugh et al., 2002). Generally, AOA and AOB are dynamic groups of marine picoplankton in the Southern Ocean, where AOA vary seasonally in the deeper waters and AOB are more variable in the upper waters. However, deeper knowledge of the distribution and composition of these ammonia-oxidizers in polar shallow coastal marine waters (up to 30 m) is still required.

Here, surface ammonia-oxidation rates and the transcriptional activity of AOA and AOB were evaluated over three consecutive summers (February–March of 2017–2019) down to 30 m in Chile Bay (62°27′6’ S; 59°40′6” W), Antarctica. We combined metagenomics, quantification of AOA and AOB *amo*A gene transcripts by RT-qPCR, and high-throughput sequencing of taxa-specific *amo*A and 16S rRNA gene transcripts to unveil the presence, abundance, and activity of the surface ammonia-oxidizer community during highly productive late-summer coastal polar ecosystem in the WAP. Our study reveals important new insights about the contribution and relevance of AOA and AOB to the WAP surface waters.

## Materials and Methods

### Study Site, Sample Collection, and Analyses

**Figure 1:**
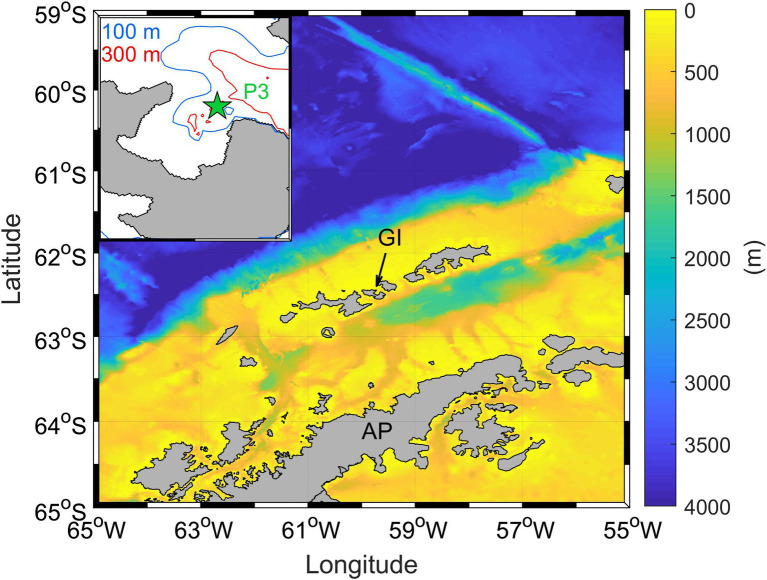
Chile bay location on the Greenwich Island (GI) in the West Antarctic Peninsula (WAP). The green star shows the P3 monitoring station in the bay.

Chile Bay (Greenwich Island, WAP) is a coastal ecosystem characterized by high variability in the phytoplankton and bacterioplankton community composition during the summer (Alcamán-Arias et al., 2018; Fuentes et al., 2019). Seawater from this bay was collected 10–12-times per summer at sampling station P3 (62°27′6” S; 59°40′6” W; Figure 1) over three consecutives late-summer periods (February to early March 2017, 2018, and 2019). Onboard a zodiac boat, seawater samples were collected at 2 m depth using a handheld pump, and at 30 m depth using Niskin bottles. Directly after seawater collection, prefiltration with a 150-μm mesh was used to exclude large organisms. At the same time a CTD instrument (SeaBird 19plus) was deployed to obtain temperature (°C) and salinity profiles. The Brunt-Väisälä frequency (cycle/h) was estimated to identify water column mixing and stratification periods during the late-summer. Nutrients [nitrate (NO_3_^−^), nitrite (NO_2_^−^) and ammonium (NH_4_^+^)], were measured at both depths for all summer periods and years. Additionally, complete seawater nutrient profiles (2, 5, 10, 20, 30, 40, and 50 m) were obtained in 2018 and 2019 for high vertical resolution. All nutrients were collected in triplicate 15 ml polyethylene flasks and frozen at-20°C until laboratory analysis *via* colorimetric determination using a AutoAnalyzer® (AA3 Seal Analytical; Caspers, 1985). NH_4_^+^ was immediately measured (in triplicate) fluorometrically (Fluorometer Turner Design AquaFluor; Holmes et al., 1999). Total chlorophyll *a* (Chl-*a*) was determined (in triplicate) by filtering 1 L of collected seawater through a 0.7-μm GF/F glass fiber filter, and then freezing the filters until analysis by acetone extraction and fluorometric measurement (Strickland and Parsons, 1972).

### Ammonia-Oxidation Rates

To obtain ammonia-oxidation rates, the isotopic ^15^N-labeled-NH_4_^+^ technique was performed in 2018 (19 February) and 2019 (19 February). Seawater samples were collected from station P3 at both 2 and 30 m depth and maintained in 2.7 L polycarbonate bottles. All bottles with seawater from 2 to 30 m, were submerged in seashore using a floating array to simulate temperature and light at surface seawater *in situ* conditions. Briefly, duplicate clear polycarbonate bottles were used to perform light incubations for the 2 m samples, meanwhile covered bottles were used to simulate darkness for the 30 m depth samples (according to photic layer depth estimations and 1% of the light penetration obtained by the Secchi disk and PAR sensor at sampling point P3, Supplementary Figure S1). For each bottle, 1.5 ml of ^15^NH_4_Cl (99% at 0.5 μmol ml^−1^) was added to obtain a final concentration of 0.5 μM ^15^NH_4_Cl. For the initial time (*t*_0_), each bottle was vigorously homogenized, and then duplicate 40 ml subsamples were collected and filtered through 0.7 μm GF/F glass fiber filters into 50 ml polyethylene tubes. After 24 h of incubation, additional 40 ml subsamples were taken from each bottle, as described above, to obtain the final time (*t*_24_). Each subsample from *t*_0_ and *t*_24_ were frozen at −20°C until further analysis at the UC Davis Stable Isotope Facility, United States. The ^15^NOx produced during the incubations (*t*_0_ and *t*_24_) was measured using the denitrifier method (Sigman et al., 2001) and the nitrification rate from atm% ^15^N values of the NO_x_ pool was calculated as described by (Christman et al., 2011) with some modifications according to the followed equation:


$$NITrate=\frac{\left[NO_{3}{}^{-}+NO_{2}{}^{-}\right]}{t}\times \frac{\left(t_{f}NOx-t_{0}NOx\right)}{\left(nNH_{4}{}^{+}-n_{0}NH_{4}{}^{+}\right)}$$

where t_f_NO_x_ and *t*_0_NO_x_ are the atm% ^15^N at the final (*t*_24_) and initial times (*t*_0_), respectively; nNH_4_^+^ is the atm% ^15^N-NH_4_^+^ enrichment at the initial time after tracer was added based on the natural NH_4_^+^ concentration and natural ^15^N abundance; n_0_NH_4_^+^ is the natural atm% ^15^N abundance obtained for Chile Bay (0.3548); and [NO_3_^−^ + NO_2_^−^] is the *in situ* inorganic nitrogen pool concentration recorded in the bay. Considering the high concentration of ^15^N-labeled NH_4_^+^ added to the incubations, isotope dilution was likely minimal (Christman et al., 2011); thus, isotopic dilution was not corrected in our estimations.

### Genomic Identification of the Nitrification Pathway

Seven gene modules of the nitrogen cycle (including nitrification), and the potential polar microorganisms involved, were investigated through metagenomic analysis for the following samples (date; depth): 2017 (21 February; 2 m); 2018 (3 March; 2 m); and 2019 (5 March; 2 m and 30 m). Duplicate 3 L volumes of seawater were obtained for the target depths at station P3 to extract and quantify nucleic acids (DNA and RNA). Using a peristaltic pump (Cole Palmer System Model no. 7553–70; 6–600 rpm), the microbial biomass was retained on 0.22 μm pore size filters (Sterivex units, Merck-Millipore). All filters (DNA and RNA) were frozen and maintained at −80°C until nucleic acid extraction. For RNA samples, the biomass collected on the Sterivex filters was preserved with 1 ml of RNAlater RNA Stabilization Solution (Ambion Inc.).

DNA extractions for metagenomic analysis were based on a previously described method (Alcamán-Arias et al., 2018) with some modifications. Briefly, filters were resuspended in lysis buffer containing xanthogenate buffer [1% potassium ethyl xanthogenate (Sigma-Aldrich, United States)]. Next, SDS was added to 1% and then the mixture was incubated at 65°C for 2 h. DNA was extracted with phenol: chloroform: isoamyl alcohol (25: 24:1) and then the residual phenol was eliminated with chloroform: isoamyl alcohol (24:1). The extracts were cleaned by precipitation overnight with cold isopropanol and then washed with 70% ethanol. The quality and quantity of the extracts were checked using a Nanodrop spectrophotometer and a Qubit® 2.0 Fluorometer (Thermo Fisher Scientific, United States) with the dsDNA-BR Assay kit. Finally, DNAse/RNAse-free 0.8% agarose gel electrophoresis was used to check the integrity.

For each sample, approximately >500 ng of total DNA was sent for Illumina sequencing on the NovaSeq 6000 platform with a read length of 150 bp (paired-end; Roy J. Carver Biotechnology Center, Illinois, United States). The quality of the metagenomic raw reads was assessed through FastQC (v0.11.9).^1^ Quality trimming of sequences was performed using Cutadapt software (v1.15; Martin, 2011), with a hard clip of the first nine bases of 5′ end and quality trimming of the 3′ end with a minimum quality of 30. Reads with ambiguous bases and low complexity sequences were removed using PRINSEQ (−ns_max_p 0-lc_method dust-lc_threshold 7; v0.20.4; Schmieder and Edwards, 2011). Paired-end quality-filtered reads were then assembled using metaSPAdes (default; v3.13.0; Bankevich et al., 2012), and subsequent ORF identification and protein prediction was performed with >500 bp contigs using Prodigal (−n-p meta; v2.6.3; Hyatt et al., 2010). Metrics of the metagenomes and the assemblies are in Supplementary Table S1.

Proteins related to seven nitrogen cycle modules (Supplementary Table S2) were searched for using hmmsearch (hmmer.org; v3.3) with previously built hidden Markov models (HMMs; Supplementary Table S2). We searched for possible nitrification enzyme orthologues in the same predicted proteins with DIAMOND (blastp-e 1e-5 --id 80 --query-cover 70; v2.0.8.146). The identified proteins were curated against the KEGG database using BlastKOALA (v2.2; Kanehisa et al., 2016). The genes that encoded the resulting proteins were used as a database target in each metagenome using Bowtie2 (v2.4.1; Langmead and Salzberg, 2012). The presence of a gene was defined by >1X coverage (depth of coverage) and > 60% of coverage breadth using “pileup.sh” from BBTools (v38.86).^2^ Thus, we assumed the presence of an entire pathway if one of the genes of the pathway was present, as described in (Nelson et al., 2016). The taxonomy of each gene was assessed through DIAMOND BLASTP against the NCBI NR database (March 2021). The taxonomy of the resulting alignments was determined using the “lowest common ancestor” (LCA) algorithm from MEGAN6 (v6_19_0; Hyatt et al., 2010) with a minimum score of 50 and default parameters. The relative abundance of nitrifying microorganisms was evaluated using two different softwares: _Metaxa2 (v2.2; Bengtsson-Palme et al., 2015) was used to identify 16S rRNA-like reads in the metagenomes, and Centrifuge (v1.0.4; Kim et al., 2016) was used to classify all reads in the metagenomes using a custom database composed by the genomes deposited in the Genome Taxonomy Database (GTDB; Méric et al., 2019). Raw paired-end reads from the marine metagenomes have been submitted to NCBI SRA under BioProject ID: PRJNA805531.

### Quantification of *amo*A Gene Transcripts of Archaea and Bacteria

The *amo*A gene was evaluated by reverse transcription quantitative PCR (RT-qPCR) to detect and quantify the active ammonia-oxidizers (AOA and AOB) discovered by metagenomic analysis. We evaluated the compatibility of the AOA *amo*A genes identified in the metagenomes and AOA *amo*A degenerate primers using the gen_primer_match_report.py program (Delmont et al., 2018). The Arch-amoA-for primer had 100% match to the identified *amo*A genes, while the Arch-amoA-rev presented between 0 and 1 mismatch.

RNA extraction was conducted from the RNA filters (duplicates) obtained as explained above. Each filter was removed from the Sterivex casing, cut into pieces using a sterile blade and then placed in a 1.5-ml tube. To each tube, 1 ml of TRIzol (Invitrogen) and ~200 μl of glass beads (0.5 mm) were added. The mixture was then subjected to two bead-beating steps of 20 s. After adding 150 μl of chloroform, the mixture was shaken for 30 s and then centrifugated for 15 min at 13,000 × *g* and 4°C. The RNA was purified from the subsequent aqueous phase using an RNA Clean and Concentrator kit (Zymo Research, United States) according to the manufacturer’s instructions. Finally, the quality and quantity of the total RNA was checked the same way as the DNA extracts explained above but using the Qubit RNA-BR Assay kit. Unfortunately, not all replicates obtained enough total RNA, therefore only one replica per day of monitoring was used.

Total RNA (normalized to 750 ng per sample) was first treated with Ambion DNase I (RNase-free; Thermo Fisher Scientific). Next, specific cDNA was synthesized from the RNA using the iScript cDNA synthesis kit (BioRad) with primers targeting the *amo*A (A and B groups of nitrifiers) genes as previously described (Tolar et al., 2016; Supplementary Table S3). Absolute quantification of *amo*A transcripts was done by RT-qPCR in a Light Cycler 480 (Roche Life Science). For each sample and year, synthetized cDNA was analyzed against plasmid calibration curves (10^2^–10^8^ copies per μl) for both AOA and AOB *amo*A genes. The thermocycler program incorporated 40 cycles of denaturing at 95°C (10 s), annealing at 58°C (20 s), extension at 72°C (30 s) and fluorescence recovery at 77°C (1 s). For each year, the external plasmid curves for both AOA and AOB (*r*^2^: >0.98; efficiency 94%–110%) were used to quantify the transcript number. Additionally, cDNA of 16S rRNA (ß-subdivision of the class Proteobacteria) gene from AOA and AOB was also synthetized as previously described by Kowalchuk et al. (1997) and Tolar et al. (2016; Supplementary Table S3). However, because at the low efficiency and slope of external plasmid curves the AOA and AOB 16S rRNA genes, the absolute quantification was not performed. Finally, specific sequencing of both *amo*A and 16S rRNA (AOA and AOB) gene transcripts were performed by Fluidigm (Illinois, United States) as described below.

### 16S rRNA and *amo*A Gene Transcript Sequencing

Illumina sequencing combined with Fluidigm 2 Step Access Array Amplification (University of Illinois Keck Center, Illinois, United States) allowed for high throughput sequencing of both the 16S rRNA and *amo*A gene transcripts from the same cDNA stock that was used for RT-qPCR. The raw AOA and AOB sequences were demultiplexed using the q2-demux plugin implemented in the QIIME2 pipeline (Bokulich et al., 2018; Bolyen et al., 2019). The paired-end sequences were trimmed and merged using DADA2 (Callahan et al., 2016) to obtain amplicon sequence variants (ASVs). Taxonomy of the 16S rRNA gene ASVs from AOB was assigned using the q2-feature-classifier, SILVA132 database (Quast et al., 2013) and the “classify consensus vsearch” method (Rognes et al., 2016). The AOB *amo*A reads were not long enough to merge the paired-end sequences due to poor sequencing quality. The AOB *amo*A gene taxonomy was obtained using BLASTN against the NCBI RefSeq genome database (January 2020) followed by the LCA algorithm implemented in MEGAN6 (v6_19_0; Hyatt et al., 2010) with a minimum score of 50 and default parameters.

### Phylogenetic Placement of AOA 16S rRNA and *amo*A Query Sequences

A reference phylogenetic tree of the *amo*A gene was first built using a curated *amo*A nucleotide database from Alves et al. (2018) and the NT NCBI database (January 2021). Reference *amo*A sequences were aligned using MAFFT (−-auto; v7.310; Katoh and Standley, 2013). The alignments were manually refined and then the phylogenetic tree was produced using iQtree (−m GTR + F + I + G4-bb 10,000-alrt 10,000; v1.6.8; Nguyen et al., 2015; Hoang et al., 2017) as described in (Alves et al., 2018). We aligned the query sequences (*amo*A ASV) to the reference alignment using MAFFT (−-keeplength –add; v7.310; Katoh and Standley, 2013) and then phylogenetically placed them into the reference tree using the EPA-ng algorithm (v0.3.; Barbera et al., 2018). The resulting tree was visualized using iTOL (Letunic and Bork, 2019).

The reference 16S rRNA phylogenetic tree was build using Nitrosopumilaceae 16S rRNA gene sequences from SILVA (release 138.1; Quast et al., 2013). The reference 16S rRNA gene sequences were aligned using MAFFT (−-auto; v7.310; Katoh and Standley, 2013). As described above, we manually refined the alignments and produced the phylogenetic tree with iQtree (−m GTR-bb 1,000-alrt 1,000; v1.6.8). MGIa 16S rRNA query sequences (ASV) were phylogenetically placed into the 16S rRNA reference tree, as described for the AOA *amo*A ASV sequences. Paired-end sequences from the AOA *amo*A and 16S rRNA genes have been deposited in the NCBI SRA under BioProject ID: PRJNA701028.

## Results

### Ammonia-Oxidation Rates and Physicochemical Characterization in Chile Bay Seawater

For the 2 m depth samples subjected to illuminated incubation, the ammonia-oxidation rates measured in the summers of 2018 and 2019 showed mean values (± standard deviation) of 68.31 ± 3.68 and 37.28 ± 6.91 nmol N L^−1^ day^−1^, respectively. However, higher rates were found for the 30 m samples subjected to dark incubation, reaching values of 267.75 ± 31.21 for 2018 and 109.38 ± 43.22 nmol N L^−1^ day^−1^ for 2019 (Table 1).

**Table 1:** Ammonia-oxidation rates recorded at 2 and 30 m depth in the surface layer of Chile Bay during the 2018 and 2019 late-summer periods.

Depth (m)	Year	Nitrificacion rate (nmol N L^−1^ day^−1^)	SD
2	2018	68.31	3.68
2	2019	37.28	6.91
30	2018	267.75	31.21
30	2019	109.38	43.22

**Figure 2:**
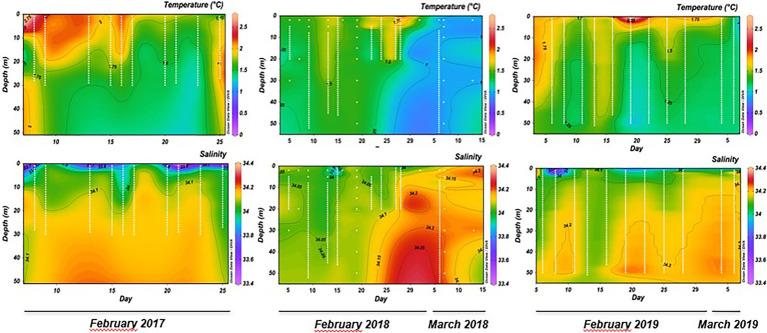
Inter-seasonal physical variables (temperature and salinity) recorded at sampling station P3 in Chile Bay over three late-summer periods (February to early March 2017, 2018, and 2019).

Generally, the ammonia-oxidation rates at 2 m were obtained along the shallow stratified column water, and high rates at 30 m were obtained under the mixed conditions. This pattern was revealed by the Brunt-Väisälä Frequency (cycles/h), which denoted short periods (4–15 days) of shallow haline stratification (salinity < 33.9) between 3 and 10 m depth (Supplementary Figure S2). Additionally, parameters, such as temperature and salinity, also showed changes under stratified or mixed conditions, where the surface layer temperature during stratification was between 1.6°C and 2.4°C (Figure 2). This surface temperature decreased to ~1.6°C during the mixing events, where increased salinity (34.1) was observed (Figure 2). Potential intrusion of temperate and saline deep water were evaluated by T-S plot (Supplementary Figure S3; Schlitzer, 2014), which showed that the first meters of the water column in the bay during the summer time monitoring period was characterized by surface slope water (SSW) partially mixed with modified circumpolar deep water (mCDW). Therefore, during the late-summer period, direct input of CDW can be excluded from this surface layer (Supplementary Figure S3). Conversely, NO_2_^−^, NO3-, and NH_4_^+^ average concentrations (Supplementary Table S4) were similar in both depths. NO_2_^−^ values remained between 0.20 and 0.28 μM, while mean NO3-concentrations were always between 19.01 and 22.84 μM. NH_4_^+^ concentrations showed values such as 0.21 and 0.35 μM not varying in depth or in periods of stratification or mixing (Supplementary Table S4). The biological variable Chl-*a* showed for the three summers (2017, 2018, and 2019) mean values twice as high at 2 m (0.55, 8.41, and 4.03 mg m^−3^, respectively) compared to 30 m (0.25, 4.93, and 2.11 mg m^−3^, respectively; Supplementary Table S4).

### Genomic Analysis of the Nitrification Pathway

From the 23 marker genes related to the seven nitrogen cycle modules (Supplementary Table S2), we could only identify nine coding genes related to the nitrate and nitrite reduction, nitric oxide reduction and ammonia oxidation modules in the metagenomes from 2017, 2018, and 2019 (Supplementary Table S5). Specifically, genes related to ammonia-oxidation were present during the summers of 2018 and 2019 (Supplementary Table S5). The taxonomy of the *amo*A and *amo*C genes indicates the presence of *Nitrosopumilus* (*amo*A and also *nir*K) and a member of the Nitrosomonadaceae family (*amo*C), thus indicating that during these two summers there were at least two groups that participated in the ammonia-oxidation process. The relative abundance of these two marker genes was very low, which helps to explain why we could not reconstruct and identify the whole ammonia oxidation operon. To evaluate the relative abundance of taxa associated with each module, we used Metaxa2 to classify 16S-like reads. For the ammonia oxidation process we could only identify 16S-like reads assigned to the family Nitrosomonadaceae and the phylum Thaumarchaeota as potential nitrifier organisms reaching <0.07% of the total bacterial and archaeal communities (Supplementary Table S6). However, analysis using all the metagenomic reads and a custom GTDB database deepened our classification of AOB and AOA. We were able to identify AOB, such as *Nitrosomonas* and *Nitrosospira* as potential ammonia oxidizers, reaching an average relative abundance of 0.046% and 0.012%, respectively, in the total community. In turn, the AOA *Nitrosopumilus* (0.132%) and *Nitrosopelagicus* (0.021%) appeared as the dominant Thaumarchaeota in all years, being more abundant in the summer of 2019 at 30 m depth (Supplementary Table S7).

### Seasonal Late-Summer Dynamics of *amo*A Gene Transcripts

**Figure 3:**
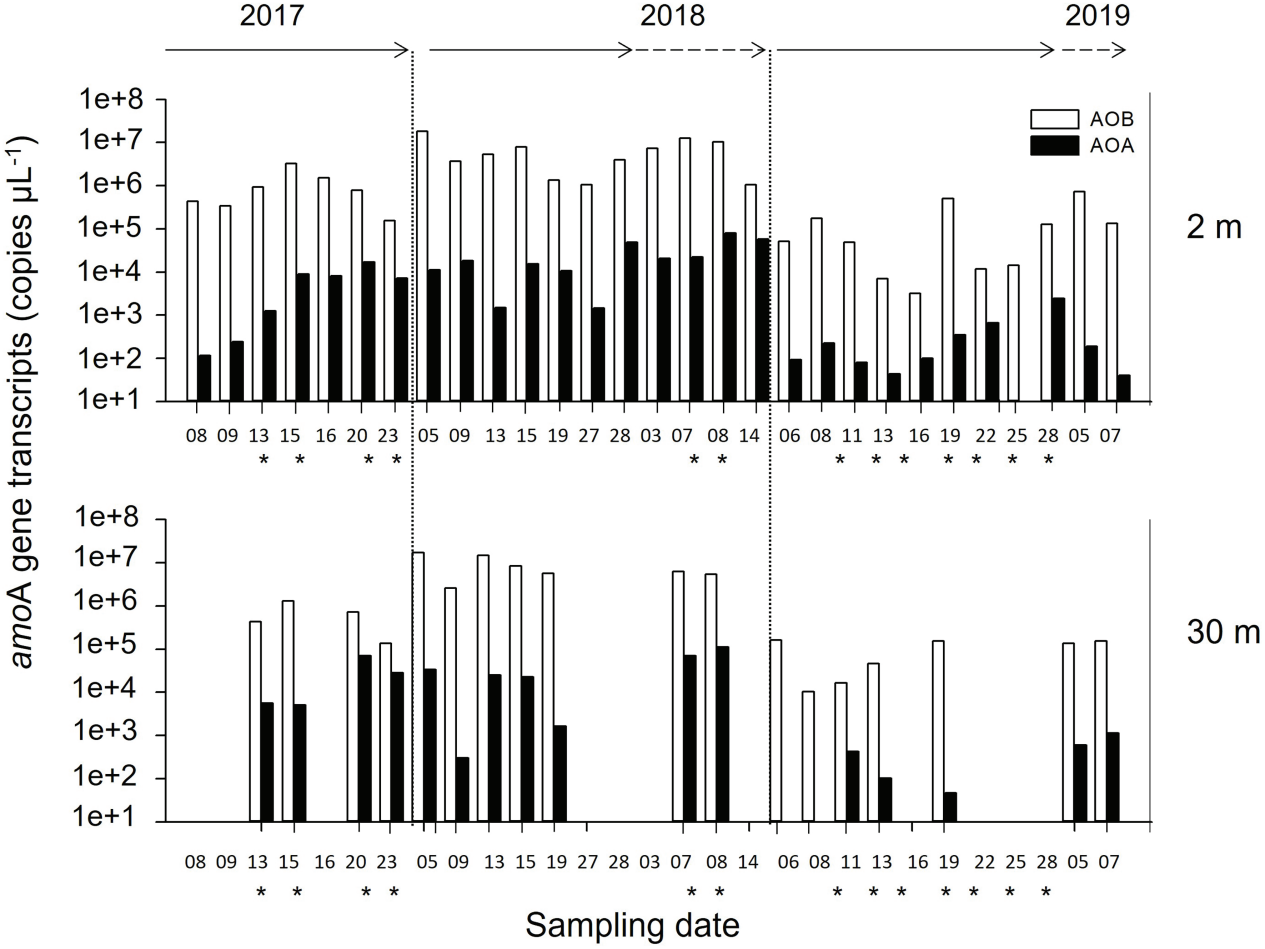
Abundance and inter-seasonal dynamics of *amo*A transcripts for ammonia-oxidizing archaea (AOA) and bacteria (AOB) recorded at 2 and 30 m depth in Chile Bay over three late-summer periods (February to early March of 2017, 2018, and 2019). White and black bars represents the number of *amo*A transcripts for AOB and AOA, respectively. The lack of bars at 30 m means the loss of samples at that depth. *Represents stratified water column events and solid and dotted arrows represent February and March, respectively.

The dynamics of both AOB and AOA *amo*A transcripts were recorded during the late-summer periods using RT-qPCR (Figure 3). The AOB *amo*A transcripts were always more abundant than AOA *amo*A at both sampling depths during all study years (Figure 3). In general, both AOB and AOA notably varied between summers at 2 m. AOB fluctuated between 3.2 × 10^3^ and 1.8 × 10^7^ copies μl^−1^, while AOA varied between 1.2 × 10^2^ and 2.0 × 10^4^ copies μl^−1^. Specifically, the highest AOB and AOA *amo*A transcripts (1.8 × 10^7^ and 8.0 × 10^4^ copies μl^−1^, respectively) were recorded in the summer of 2018 relative to the other 2 years. At 2 m, the AOB *amo*A transcripts were three orders of magnitude higher than the AOA *amo*A transcripts in 2017 and 2019 (Student’s *t*-test, *p* < 0.05), but only two orders higher in 2018 (Student’s *t*-test, *p* < 0.002). At 30 m, the AOB transcripts were two orders of magnitude higher than AOA in 2018 and 2019 (Student’s *t*-test, *p* < 0.05), but only one order of magnitude higher in 2017 (Student’s *t*-test, *p* < 0.08).

### Identity and Phylogeny of the Active Ammonia-Oxidizer Community

**Figure 4:**
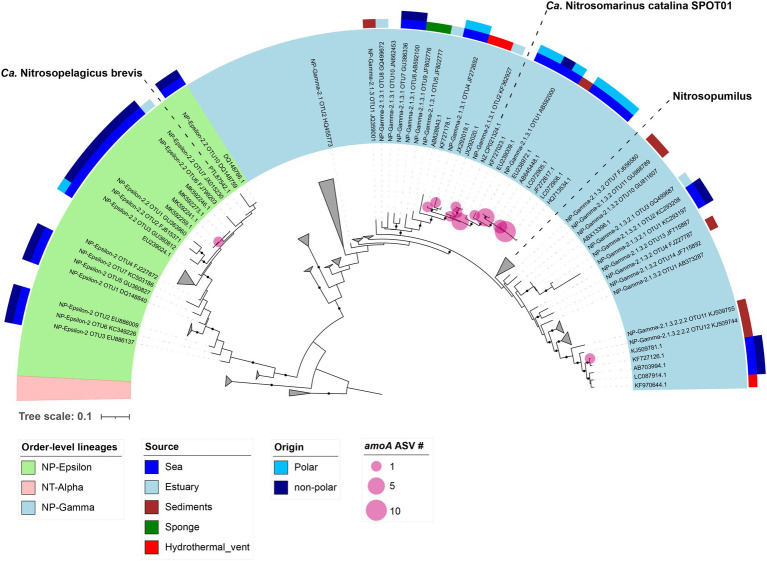
Phylogenetic tree representing Archaeal *amo*A relatives. The green and blue rings refer to NP-ε and NP-γ orders-level lineages, respectively. The colored upper rings are related to the source, origin and latter clade affiliation of the AOA nitrifiers. Pink circles over nodes indicate AOA *amo*A ASVs number to each lineage.

The taxonomic identity and phylogeny of the AOA (Figure 4) and AOB *amo*A (Supplementary Figure S4) and AOA 16S rRNA genes (Supplementary Figure S5) were resolved by sequencing the cDNA of the samples from each year and depth. Taxonomic analysis of the AOB 16S rRNA genes showed ubiquity in all years and depths for AOB related to members of Betaproteobacteria, such as *Nitrosomonas*. Additionally, representatives of the family Methylophilaceae and the genus *Nitrosospira* were detected at low levels in 2017 and 2018 (Supplementary Figures S4a,d). The AOB *amo*A genes were only taxonomically identified in the 30 m samples, specifically being related to nitrifier bacteria of the Nitrosomonadaceae family; however, it was impossible to obtain the taxonomic identity of those at 2 m due to poor sequence quality.

The AOA *amo*A transcripts were distributed between the different years and depths (Supplementary Figures S4c,f). The relative abundances of these archaeal *amo*A ASV revealed the ubiquity and persistence of the clade NP-Gamma-2.1.3.1. Additionally, ASV affiliated to the clade NP-Gamma-2.1.3.1 accounted for >90% of *amo*A ASVs relative abundance in 2017 at 2 m and < 5% at 30 m in 2018, whereas *amo*A ASVs from clades NP-2.1.3.2 and NP-Epsilon-2.2 were accounted for less than 4% in 2018 and 2017 (Supplementary Figures S4c,f). The analysis of the archaeal 16S rRNA ASVs showed the presence of a third archaeon that was related to *Ca.* Nitrosarcheum, with four ASVs positioned in a clade composed of *Ca.* Nitrosarcheum 16S rRNA sequences (Supplementary Figures S4b,e). During the three austral summers, we identified AOA 16S rRNA ASVs related to *Ca.* Nitrosopelagicus, *Ca.* Nitrosopumilus and *Ca.* Nitrosarcheum, meanwhile AOA *amo*A ASVs were mainly belonged to the order-level lineage NP-2.1 represented by *Ca.* Nitrosopumilus (Supplementary Figures S4b,e).

Phylogenetic reconstructions demonstrated that most of the AOA *amo*A ASV retrieved in Chile Bay clustered together with sequences from other marine seawaters and sediments of not only polar but also temperate oceans (Figure 4). In particular, ASVs from the NP-Epsilon-2.2 clade (light green, Figure 4) clustered with sequences from family NP-ε 2.2, related to *Ca.* Nitrosopelagicus brevis and other sequences from non-polar regions. *amo*A ASVs (light blue, Figure 4) positioned into family NP-γ 2.1 and were closely related to the genera *Nitrosopumilus* and the newly described *Ca.* Nitrosomarinus, clustering with polar and non-polar marine sequences.

## Discussion

### Ammonia-Oxidation Rates in the Surface Waters of Chile Bay

Nitrification is known as an important process that provides regenerated NO_3_^−^ to the oceanic euphotic zone (Raimbault et al., 1999; Raimbault and Garcia, 2008; Fernandez and Farías, 2012). However, available knowledge of this process is very limited for the South Ocean. Chile Bay, located on the Greenwich Island, WAP, was monitored over three consecutive late summers to quantify the ammonia-oxidation rates and reveal the principal AOA and AOB involved in this process.

On a seasonal timescale between February and early March, our oceanographic results demonstrate that Chile Bay is characterized by a warm surface layer due to less wind, continuous solar radiation and few cloudy days during the summer. Nevertheless, the shallow haline stratified layer (up to 15 m) is interspersed below this depth with periods of a mixed photic layer, which is probably due to sporadic strong winds (Fuentes et al., 2019). The haline stratification can be explained by the effect of shallow glacier plumes characterized by low salinity (<33.9) and coastal glacier melt events that have recently been demonstrated in this bay (Alcamán-Arias et al., 2021). Under these oceanographic summer conditions, we detected high ammonia-oxidation rates (267.75 nmol N L^−1^ day^−1^) in mixed waters and under the dark condition; however, the lowest rates were obtained from the superficial illuminated condition (Table 1). In addition, the ammonia-oxidation rates observed in this study were similar to those previously reported for the WAP during the winter (Tolar et al., 2016) but were higher than those reported for the oxycline in the Chilean coastal zone (Fernandez and Farías, 2012), the equatorial Pacific and the Arctic Ocean (Shiozaki et al., 2016). These rates highlight the presence of nitrifying organisms, such as ammonia-oxidizers, in the shallow surface layer. This is reinforced by the fact that ammonia-oxidizers have been previously detected in Antarctic Surface Water (0.5–1.5°C, S < 33.8; 0–150 m) in the WAP (Tolar et al., 2016), conditions that are in line with the recorded temperature and salinity in the surface water of Chile Bay.

Knowing that ammonia-oxidation occurs in the surface layer of Chile Bay, metagenomic analysis was performed for 23 marker genes from seven nitrogen cycle modules, including ammonia-oxidation. The results reveal the presence of *amo*A (in 2019) and *amo*C (in 2018) genes, associated with the presence of the genus *Nitrosopumilus* and a member of the Nitrosomonadaceae family, respectively, indicating that during these two late summers there was at least one of these taxa involved in the ammonia-oxidation process. However, because the relative abundance of these two marker genes was very low, it was not possible to reconstruct and identify the entire ammonia oxidation operon. Therefore, further analysis is necessary when as in this study genes such as these were determined with a depth of coverage >1X and a breadth of coverage >60%.

### Active Ammonia-Oxidizers in the Summer Surface Layer

Quantification of specific AOA and AOB *amo*A genes allowed to reveal the presence of this community in surface waters, with a higher abundance of AOA *amo*A over AOB *amo*A during the late summer above 30 m depth. However, the AOB *amo*A transcripts were 2 or even 3-orders of magnitude higher than those of AOA *amo*A at 2 m, while AOA tended to increase at 30 m. In addition, the taxonomic identity of AOB obtained in our metagenomes analysis was confirmed by AOB 16S rRNA transcripts sequencing, which revealed that *Nitrosomonas* was the most abundant AOB in Chile Bay. However, due to the high similarity of the ASV sequences to the SILVA database representatives, a robust description by phylogenetic reconstruction of *Nitrosomonas* sequences obtained was not carried out. While *Nitrosomonas* has been observed in the coastal Artic Ocean (Hollibaugh et al., 2002; Christman et al., 2011), it reportedly accounts for <3% of the nitrifier community in the WAP (Tolar et al., 2016). In Chile Bay, the relative abundance within the total metagenomic reads denotes that AOB (*Nitrosomonas* and *Nitrosospira*) represent up to 0.066% of the bulk community (Supplementary Table S7).

The number of AOA *amo*A transcripts recorded in Chile Bay were comparable to previous WAP studies of the deeper layer, being only one order of magnitude lower than those reported at 70–125 m depth (Tolar et al., 2016). Nevertheless, our results support previous reports in which the archaeal relative abundance significantly decreased during the summer at the surface (1%–2% of the prokaryotic population) but remained high (5%–17%) below 50 m (Murray et al., 1998; Church et al., 2003). Altogether, *amo*A gene transcripts for both AOB and AOA detected in Chile Bay above 30 m in the photic layer during the summer has not been previously reported in WAP studies (Kalanetra et al., 2009; Tolar et al., 2016).

Previous studies in the area have also reported a high abundance of Thaumarchaeota *amo*A transcripts and ammonia-oxidation rates in the colder and saltier Winter Water layer (50–150 m; Tolar et al., 2016). As the dominant circulation in Chile Bay is thermohaline and influenced by the topography (Cornejo and Arcos, 1990), we suggest that thermohaline variability associated with mixing could represent the physical parameters controlling nitrifier abundance and dynamics inside the bay during the summer time.

The *in situ* ammonium concentration detected in Chile Bay would be enough to support the activity of ammonia-oxidizers under a highly productive summer period. In addition, the potentially intense competition between nitrifiers and autotrophic phytoplankton during the summer period could represent another negative factor affecting ammonia-oxidizers abundance. However, this has not been robustly evidenced in our study regardless of the high Chl-*a* concentration detected, which is typical for phytoplanktonic bloom events in this bay during the late summer (Alcamán-Arias et al., 2018). Light has also been proposed as another environmental factor affecting the vertical distribution of transcriptional activity of the marine AOA and AOB nitrifier community (Shiozaki et al., 2016). In polar regions, such as the Arctic Ocean, it has been suggested that sea ice reduction will increase light levels, thereby suppressing nitrification and altering the composition of inorganic nitrogen (Shiozaki et al., 2019). In Chile Bay, the high AOA and AOB transcriptional activity at high light exposure at 2 m was surprising, since it is known that AOA are more sensitive to photoinhibition than AOB (Merbt et al., 2012; Qin et al., 2014; Lu, 2020). Validation of our Secchi disk photic layer depth (PLD) using a PAR sensor demonstrated an average difference of 4 m, corroborating the incident light detected in this study where the 1% of light was until 20 m depth. Accordingly, other environmental factors that could attenuate this light penetration, such as suspended organic matter and high photosynthetic activity, as well as the metabolic light adaptation of these microorganisms (Luo et al., 2014), now need to be addressed.

### Phylogenetic Relation of Chile Bay Ammonia-Oxidizers

Our analysis also revealed the phylogenetic association of the AOA *amo*A transcripts in Chile Bay to the surface water-related families NP-ε 2.2 and NP-γ 2.1 (Alves et al., 2018). These families have been previously related to the surface Cluster A and the Shallow clade (Hallam et al., 2006; Beman et al., 2008; Kalanetra et al., 2009), which also dominate in the central Arctic Ocean (Kalanetra et al., 2009), as well as to abundant clades from the surface of the Amundsen Sea (Alonso-Sáez et al., 2011) and those of the WAP region (Tolar et al., 2016). The phylogenetic affiliation of the nitrifier community identified here suggests a selected presence in these polar surface waters, according to the permanent presence of the SSW without deeper water mass intrusion to the surface (Supplementary Figure S3). Thus, this suggests that the nitrifier community did not come from deep waters as a result of mixing events or from the putative intrusion of warm and fresh SSW mixed with the mCDWs (Huneke et al., 2016; Moffat and Meredith, 2018). However, new insights into this nitrifier community from the deep ocean will be necessary to confirm this hypothesis.

Despite the low diversity of ammonia-oxidizers recorded here, which were only related to *Ca.* Nitrosopelagicus, *Nitrosopumilus*, *Ca.* Nitrosomarinus, *Ca.* Nitrosarcheum and *Nitrosomonas*, these findings expand the distribution of ammonia-oxidizing prokaryotes to the surface photic layer above 30 m in coastal polar regions.

Our approach confirms that ammonia-oxidation is a key biological process in the upper coastal polar seawater. Considering that phytoplanktonic bloom events are a recurrent summer phenomena in this bay, together with high heterotrophic bacterial activity that assimilates other forms of nitrogen (Alcamán-Arias et al., 2018), the activity of the nitrifying community in the surface layer would play an important role in the availability of nitrite and nitrate regenerated at the surface by compensating the balance of the downward flux of POM sinking to the deep layers, and also by the use of nutrients recycled from POM by phytoplankton.

## Data Availability Statement

The datasets presented in this study can be found in online repositories. The names of the repository/repositories and accession number(s) can be found in the article/Supplementary Material.

## Author Contributions

MA-A wrote the original draft. MA-A, GT, MT, and EB collected field samples, performed experiments, and processed the CTD and nutrients samples. MA-A, BD, and LF reviewed and edited the manuscript and provided funding acquisition and resources. MA-A and JC-A processed the genomic sequencing data and phylogenetic reconstruction. All authors contributed to the article and approved the submitted version.

## Funding

This work was supported by the grants ANID/FONDECYT/INACH N3170807, ANID/FONDAP 15110009, and ANID/PFCHA/Doctorado Nacional/2017 21170561.

## Conflict of Interest

The authors declare that the research was conducted in the absence of any commercial or financial relationships that could be construed as a potential conflict of interest.

## Publisher’s Note

All claims expressed in this article are solely those of the authors and do not necessarily represent those of their affiliated organizations, or those of the publisher, the editors and the reviewers. Any product that may be evaluated in this article, or claim that may be made by its manufacturer, is not guaranteed or endorsed by the publisher.

## Footnotes

^1^ https://www.bioinformatics.babraham.ac.uk/projects/fastqc/

^2^ https://sourceforge.net/projects/bbmap/
