# Supplementary material for: Surface Ammonia-Oxidizer Abundance During the Late Summer in the West Antarctic Coastal System
Source: Front Microbiol. 2022 Mar 25;13:821902. doi: 10.3389/fmicb.2022.821902 (PMC8992545; doi:10.3389/fmicb.2022.821902)
Supplement: Supplementary file 1 [file Data_Sheet_1.zip › Supplementary Materials Captions.docx]

**Supplementary data**

**Table S1**. Assembly metrics of Chile Bay metagenomes for 2017, 2018 and 2019 late summers.

**Table S2**. Hidden Markov Models database used for annotation of nitrogen cycle metabolic marker genes.

**Table S3**. Primers targeting the 16S rRNA and *amo*A genes used for RT-qPCR and sequencing.

**Table S4**. Nutrients and chlorophyll a concentration at 2 and 30 m in Chile Bay seawater during February and early March of 2017, 2018, and 2019.

**Table S5**. Abundance in counts per million of nitrogen cycle related enzymes and their taxonomy.

**Table S6.** Relative abundance (%) of taxa related to the nitrogen cycle determined by Metaxa2 (SILVA128 database).

**Table S7.** Relative abundance (%) of taxas related to the nitrogen cycle determined by Centrifuge (GTDB-custom database).

**Figure S1**. Photic Layer Depth (PLD) observed in Chile Bay during late summer. The dotted grey line indicates the 1% of light penetration according to the Pearson correlation (α =0.05) between the Secchi disk and PAR sensor for summer 2018. Solid and dotted arrows represent February and March, respectively.

**Figure S2**. Brünt-Vaisälä Frequency determined during late summers of 2017, 2018, and 2019 in Chile Bay. Shallow stratified and mixed water column periods were observed during February and early March each year.

**Figure S3**. Characterization of different water mass densities down to 50 m depth in Chile Bay through T-S representation of three summer seasons (2017, 2018 and 2019). SSW: Surface Slope Water; uCDW: upper Circumpolar Deep Water; mCDW: modified Circumpolar Deep Water.

**Figure S4**. Relative abundance of AOA and AOB at 2 and 30 m. AOB 16S rRNA (a, d), AOA 16S rRNA (b, e), and AOA *amo*A (c, f) transcripts at inter-seasonal scales in Chile Bay for the different depths.

**Figure S5**. Phylogenetic reconstruction of AOA 16S rRNA recorded for 2017, 2018 and 2019 late summers in Chile Bay. Pink circles over nodes indicate AOA 16S rRNA ASVs number. The black doted over nodes represent >90% Ultrafast bootstrap support and >80% Sh-aLRT branch support.
