# Supplementary figures and images for: Surface Ammonia-Oxidizer Abundance During the Late Summer in the West Antarctic Coastal System
Source: Front Microbiol. 2022 Mar 25;13:821902. doi: 10.3389/fmicb.2022.821902 (PMC8992545; doi:10.3389/fmicb.2022.821902)

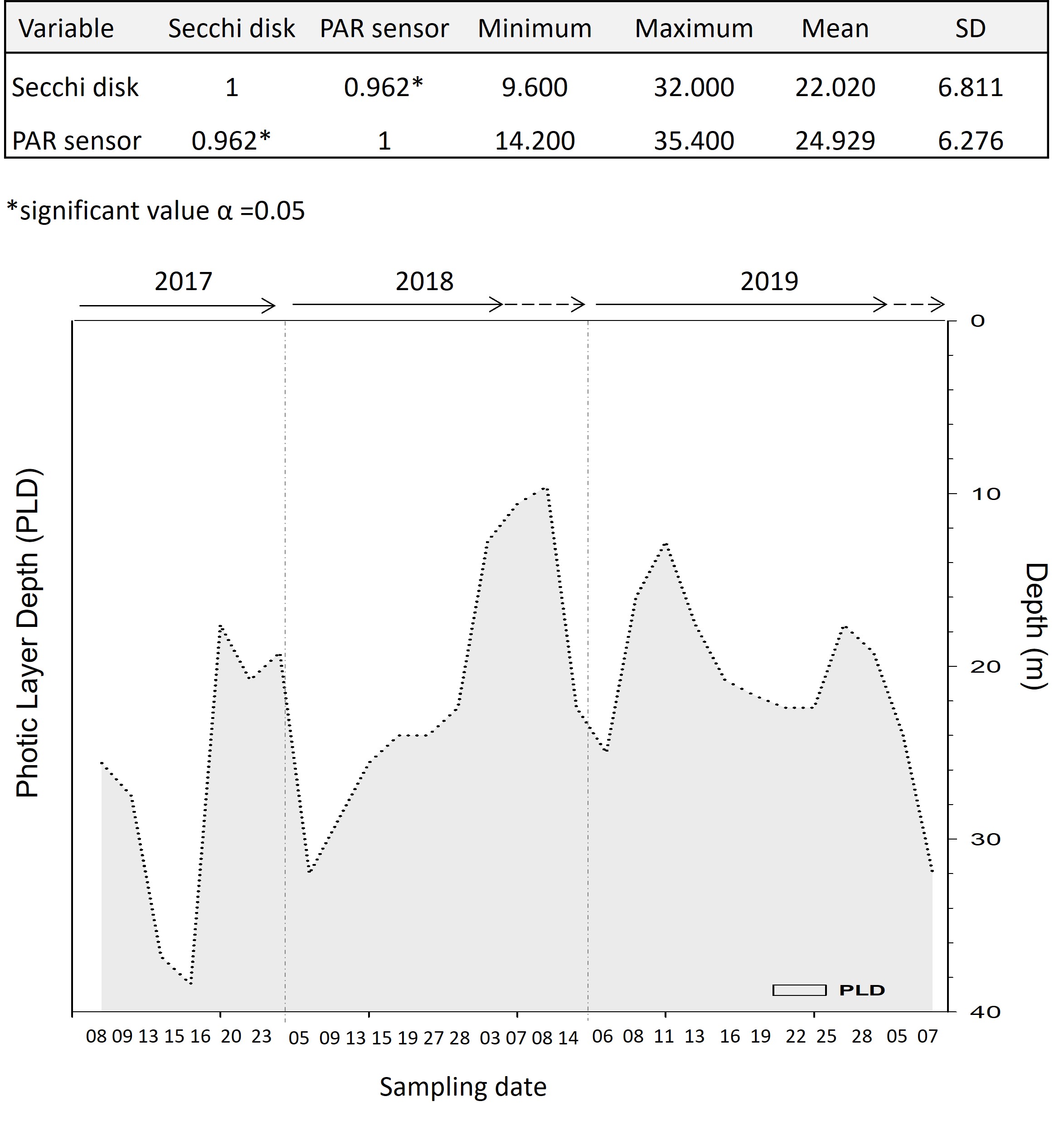

Supplement: Supplementary file 1 [file Data_Sheet_1.zip › Figure S1.JPEG]

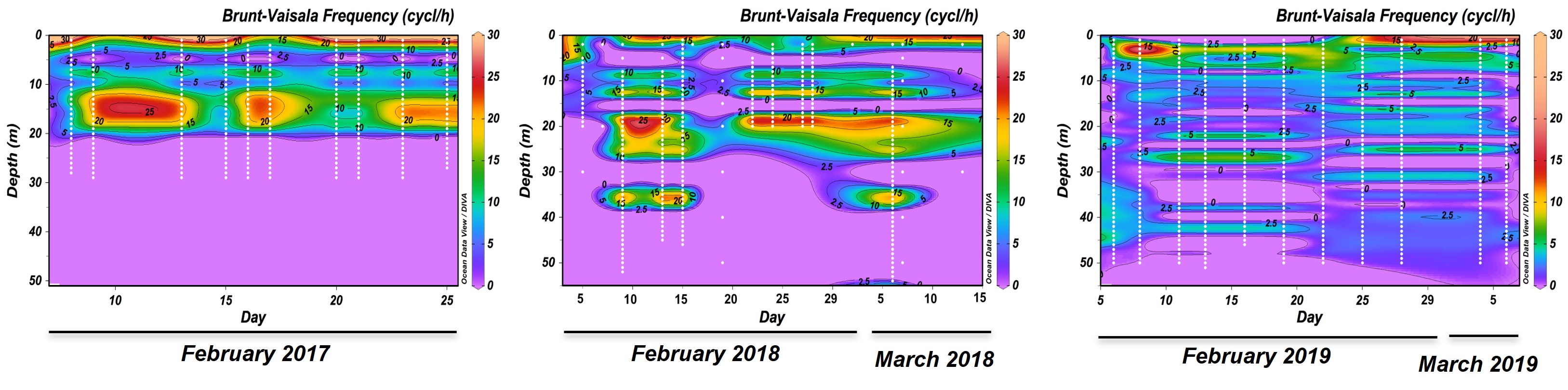

Supplement: Supplementary file 1 [file Data_Sheet_1.zip › Figure S2.JPEG]

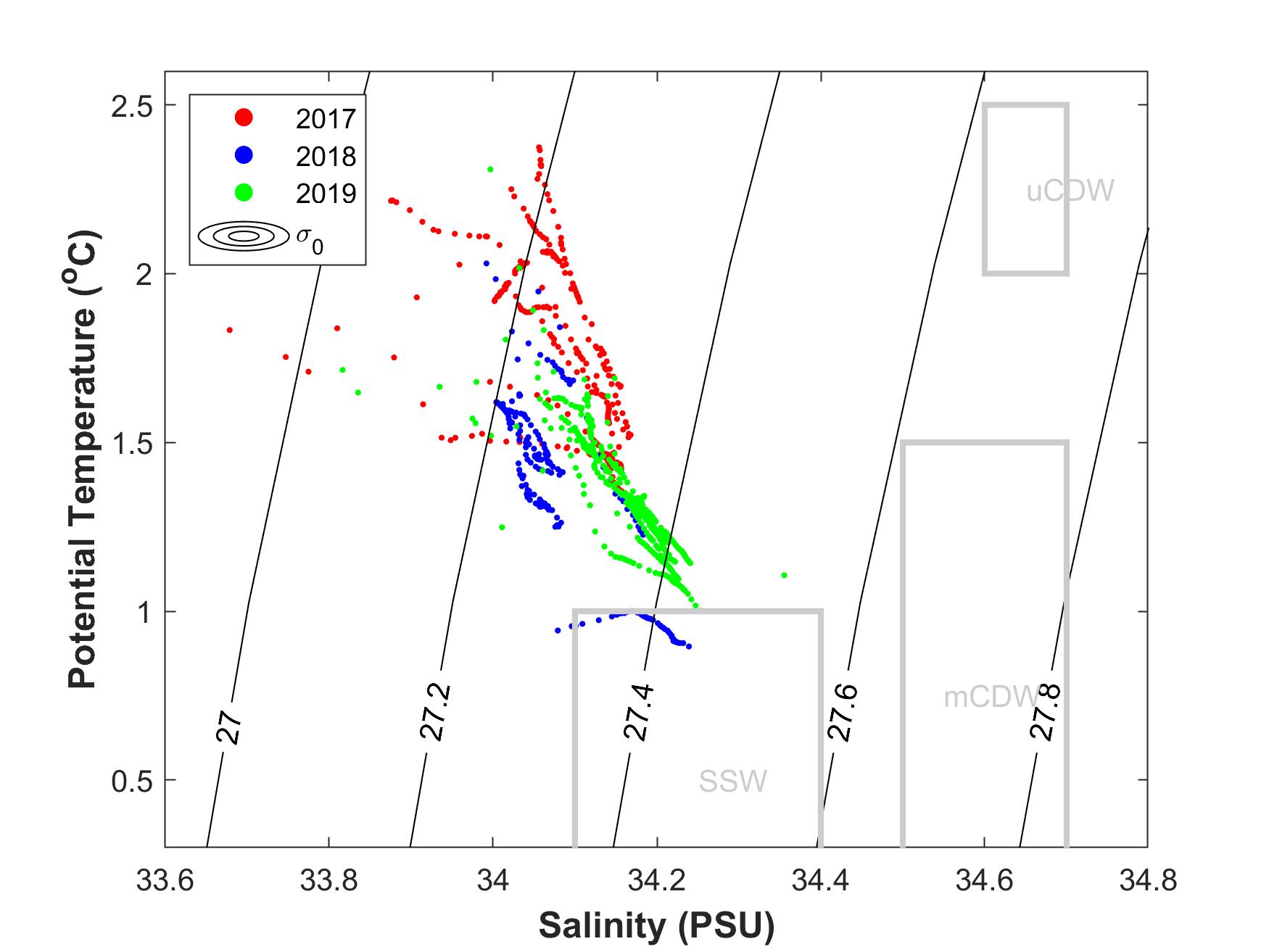

Supplement: Supplementary file 1 [file Data_Sheet_1.zip › Figure S3.JPEG]

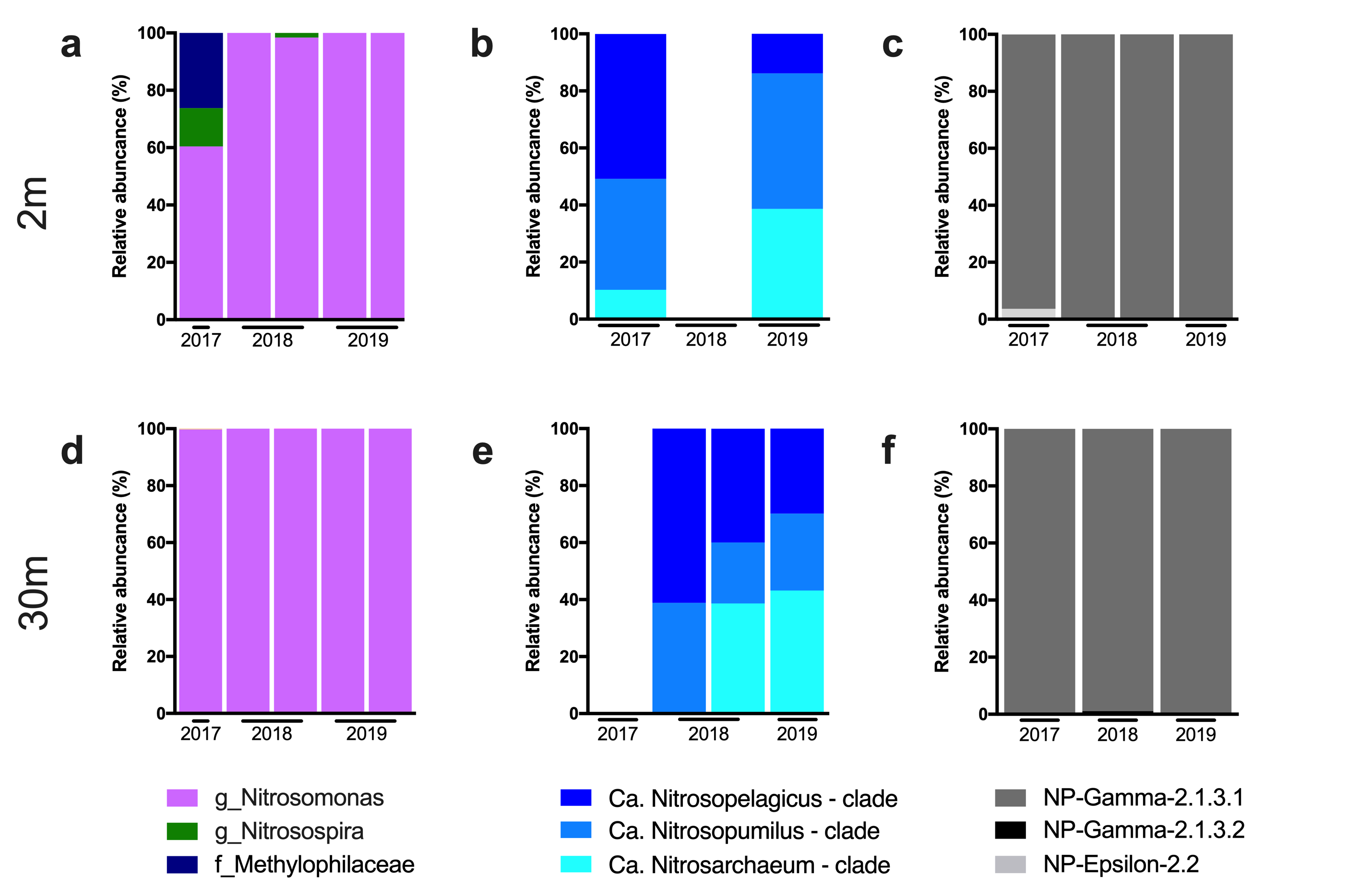

Supplement: Supplementary file 1 [file Data_Sheet_1.zip › Figure S4.JPEG]

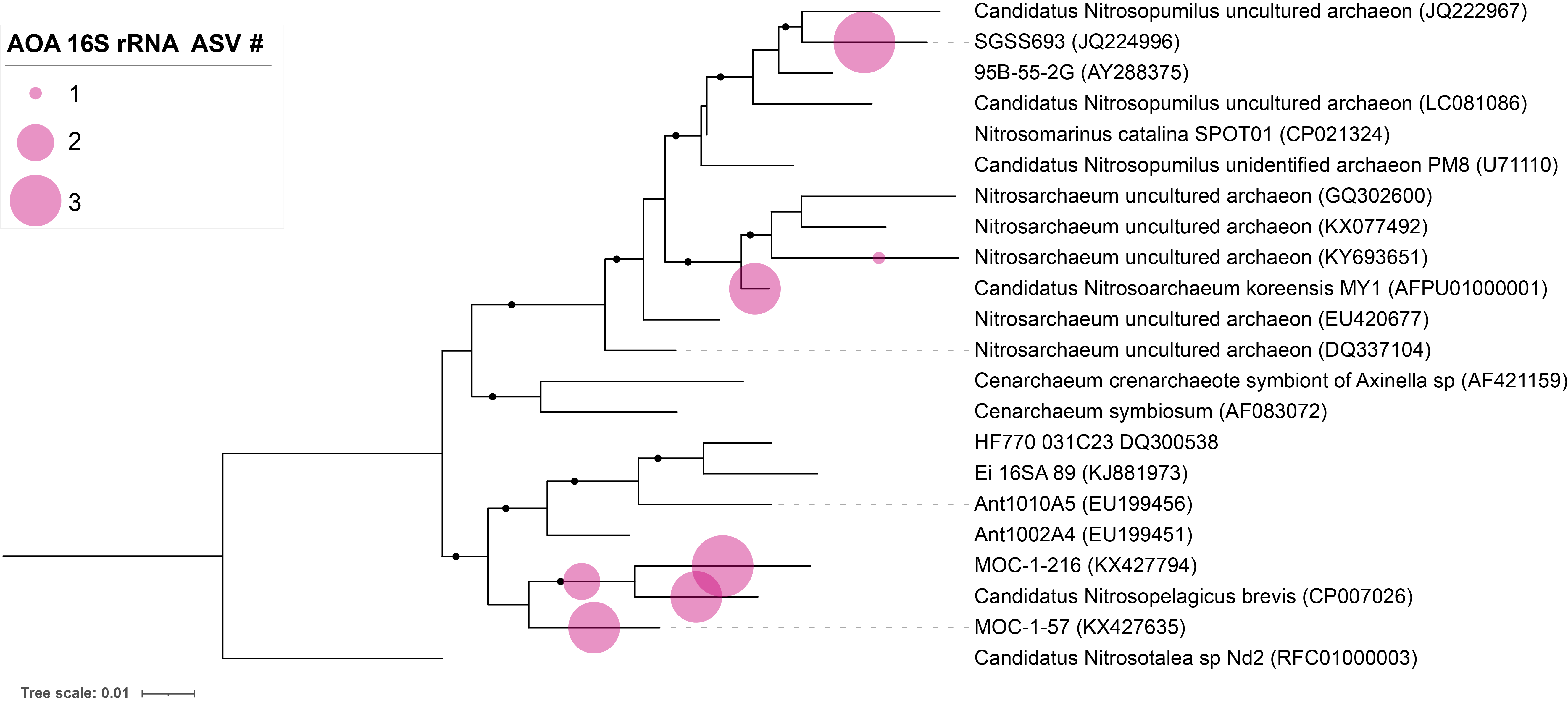

Supplement: Supplementary file 1 [file Data_Sheet_1.zip › Figure S5.JPEG]
